# Impact of Vision Loss on Visual Function Among Elderly Residents in the “Home for the Aged” in India: The Hyderabad Ocular Morbidity in Elderly Study

**DOI:** 10.1167/tvst.9.13.11

**Published:** 2020-12-07

**Authors:** Srinivas Marmamula, William Mitchell, Nazlee Zebardast, Joseph Locascio, Navya Rekha Barrenkala, Thirupathi Reddy Kumbham, Satya Brahmanandam Modepalli, Rohit C. Khanna, David S. Friedman

**Affiliations:** 1 Allen Foster Community Eye Health Research Centre, Gullapalli Pratibha Rao International Centre for Advancement of Rural Eye Care, L V Prasad Eye Institute, Hyderabad, India; 2 Brien Holden Institute of Optometry and Vision Science, L V Prasad Eye Institute, Hyderabad, India; 3 Wellcome Trust/Department of Biotechnology India Alliance, L V Prasad Eye Institute, Hyderabad, India; 4 School of Optometry and Vision Science, University of New South Wales, Sydney, Australia; 5 Massachusetts Eye and Ear, Harvard Medical School, Harvard Medical School, Department of Ophthalmology, Boston, MA, USA; 6 Massachusetts General Hospital, Boston, MA, USA

**Keywords:** elderly, residential care, India, visual functions, visual impairment

## Abstract

**Purpose:**

The purpose of this study was to report the association between visual impairment (VI) and self-reported visual difficulty among the elderly in residential care using the Indian Vision Functioning Questionnaire (IND-VFQ-33) psychometrically validated questionnaire.

**Methods:**

Participants aged ≥ 60 years were recruited from 41 homes in Hyderabad in South India. All participants underwent detailed eye examination and interviews. Self-reported visual function was assessed using the IND-VFQ-33 questionnaire. Factor Analysis and Item Response Theory (IRT) models were used for analysis. Multivariable regression models were used to investigate associations between derived global difficulty scores versus severity and causes of VI. Presenting visual acuity worse than 6/18 in the better eye was considered as VI.

**Results:**

In total, 867 elderly participants completed the INDVFQ-33. Two latent traits (“daily activities” and “visual symptoms”) were identified on factor analysis, each with uniquely loading questions. Participants with VI reported significantly higher daily activities difficulty (6 points higher) and visual symptoms difficulty (1.7 points higher) than those without VI (*P* < 0.05). Those with cataract reported the highest daily activities and visual symptoms difficulty (7.6 points and 2.2 points higher, respectively, *P* < 0.05). Greater severity of VI was associated with increased self-reported difficulty for both factors, and for all causes of VI.

**Conclusions:**

We present a psychometrically validated visual questionnaire particularly suited to older adults in residential homes. We show a significant association between cause/severity of VI and difficulty with daily activities and visual symptoms after adjusting for sociodemographic and medical factors.

**Translational Relevance:**

Understanding the impact of vision loss on visual functions in the elderly will help in planning and resource allocation for developing early intervention programs for the elderly.

## Introduction

Visual impairment (VI) affects over 253 million people worldwide, disproportionately impacting low-middle income countries, and older age groups.^1^ There is a particularly high prevalence of VI among older age groups living in residential aged care subsequently affecting visual-related function and quality of life.^2^^,^^3^ The impact of VI on visual-related function among older age groups in residential care has been previously reported in several studies from developed countries,^4^^–^^6^ however, studies from low-middle income countries with substantially larger populations like India are currently limited. Given India's growing elderly population^7^ and increasing number of residential care homes,^8^ an understanding of the effect of VI on visual and physical-function in elderly residential-care populations is becoming increasingly important.

The Hyderabad Ocular Morbidity in Elderly Study (HOMES) study was conducted to assess the burden of vision loss in elderly populations in residential care in Hyderabad, India.^9^ The study reported that 30.1% of the elderly in residential care suffer from vision loss, and notably, most VI was either preventable or treatable.^2^ The current paper aims to report the association between VI and self-reported visual function in an elderly population living in residential care in Hyderabad, India.

The current study initially applies psychometric validation techniques (Factor Analysis, Item Response Theory [IRT], and Differential Item Functioning [DIF]) to identify which latent traits are being assessed by the Indian Vision Function Questionnaire (IND-VFQ-33), which questions most suitably assess each of those traits, and calculate adjusted individual visual difficulty scores from self-reported questionnaire data. Comparisons of these adjusted scores are subsequently undertaken by cause and severity of VI, to demonstrate the utility of our approach and to understand which conditions and severity of VI most substantially contribute toward self-reported functional difficulty in an elderly residential care population in India.

## Methods

### Study Population and the IND-VFQ-33 questionnaire

In total 1182 participants from the HOMES study cohort were considered for participation.^2^ Initially, 98 (8.3%) participants were excluded for cognitive deficit (Mini-Mental State Examination [MMSE] score of 20 or lower), and a further 217 (18.4%) were unable to participate due to other medical issues and excluded. The IND-VFQ-33 was administered to the remaining 867 (73.4%) participants. The HOMES study design and procedures were approved by the Institutional Review Board of the Hyderabad Eye Research Foundation, India. The study was conducted in adherence to the Declaration of Helsinki. All participants provided written informed consent expressing their willingness to participate in the study.

The IND-VFQ-33 is a 33-item questionnaire instrument developed and validated in India^10^^–^^12^ and was administered to participants in the present study. The questionnaire assesses four visual dimensions: mobility, activity limitation, psychosocial impact, and visual symptoms.^10^^–^^12^ Questions 1 to 22 in the IND-VFQ-33 are scaled on a 5-point Likert scale, and the remaining 11 questions scaled on a 4-point scale. Options 1 to 4 on both scales are identical, and only option 5 for questions 1 to 22 differs (“cannot do this because of my sight”). Questions 1 to 22 also have a sixth option (“cannot do this for other reasons”), which was treated as a missing value for the present study. A higher score on the scale represents a higher degree of difficulty. All IND-VFQ-33 questionnaires were administered to participants by trained investigators.

### Clinical Examination and Interviews

All the participants underwent visual acuity assessment for distance and near vision under ambient light as described in our previous publications.^2^^,^^9^ VI was defined as presenting distance VA worse than 6/18 in the better eye. VI was further subdivided into moderate VI (6/18 to 6/60 in the better eye), severe VI (6/60 to 3/60 in the better eye), or blindness (worse than 3/60 in the better eye). Causes of VI were classified as cataract, uncorrected refractive error, or other causes (including age-related macular degeneration, diabetic retinopathy, posterior capsular opacification, and others). Refraction, slit-lamp examination, and fundus imaging were completed for all participants. Before eye examinations, all participants were interviewed by a trained investigator using pre-coded questionnaires as described in our previous publications.^9^ In brief, the nonclinical protocol included administration of questionnaires by the trained investigators. These questionnaires included personal, sociodemographic, ocular and systemic history, the Indian Visual Function Questionnaire (IND-VFQ-33),^12^^,^^13^ the Patient Health Questionnaire (PHQ9),^14^ and the MMSE questionnaire.^15^

### Psychometric Validation of the IND-VFQ-33 Questionnaire

Factor Analysis, IRT, and DIF validation techniques were implemented to assess psychometric properties and to modify and rescale participants’ responses to the IND-VFQ-33 questionnaire.

### Factor Analysis

**Figure 1.**
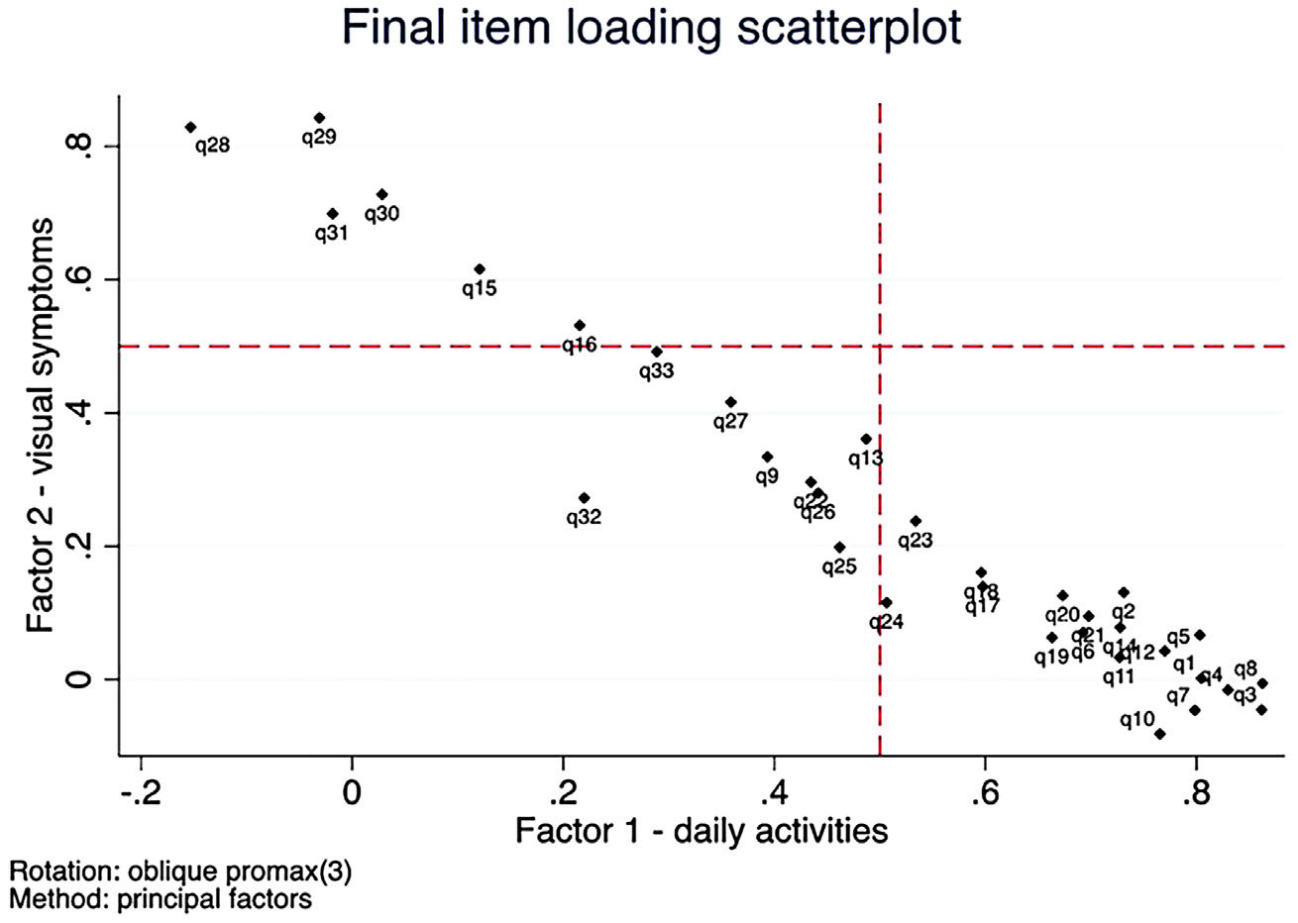
Plot of preferential and substantial item loadings in final rotated factor analysis.

Factor analysis is a psychometric method used to identify the presence and nature of latent traits (henceforth referred to as “factors”) underlying observable participant responses.^16^^–^^19^ Exploratory factor analysis analyzes the correlations of responses to questions (henceforth referred to as “items”) to identify factors, on the assumption that unique patterns of responses suggest which factors are likely being assessed, and which items relate to those factors (and to what degree)^20^.^20^ Initial, unrotated factor analysis is used to identify the number of factors present; determining if the questionnaire is unidimensional (where a single latent trait is being measured), or multidimensional (multiple latent traits measured) based on various criteria. The criteria for the present study are the “loading” associations of the items with the factors (considered substantive if > 0.5), the associated eigenvalues (variance) for each factor (considered substantive if > 1), a “screeplot” of the eigenvalues (selecting factors above the asymptote point), and a “parallel analysis,”^19^^,^^20^ which compares obtained eigenvalues to the 95th percentile of distributions of eigenvalues based on random data. The short-listed number of factors is then specified in a subsequent factor analysis in which the loadings are “rotated” to a substantively meaningful and parsimonious solution of items loading on each factor (Fig. 1). We used an “oblique” rotation method (Promax) that allowed factors to be moderately correlated if empirically indicated.^21^ Finally, Cronbach alpha coefficients (an index of internal consistency reliability and unidimensionality) for items loading on each factor^22^ were calculated.

A pairwise deleted correlation matrix was used initially to handle missing data for the factor analyses, using each item response, irrespective of missingness of other items in a respondent's questionnaire. All items with > 20% missing values were removed from the final list of factors. In addition, items were removed if they were considered either too ambiguous (i) in wording (with subsequent poor factor loading < 0.5), and/or (ii) in which factor on which they predominantly loaded (simultaneously loading more than one factor).

### Item Response Theory 

IRT models validate how well individual questionnaire items discriminate between participants of differing ability, and how clearly those differences are reflected by item responses. The specific class of IRT model used for the present study was a graded response model (GRM),^23^ which applies the principles of traditional dichotomous unidimensional IRT models to polytomous or ordinal data (like the IND-VFQ-33).^24^ The GRM calculates a series of dichotomous probabilities for each option on the polytomous Likert scale, and the subsequent level of ability (or visual “disability” in this case) a respondent would need to be most likely to answer at a certain response level on the Likert scale.^23^^,^^24^

The GRM calculates individual item discrimination values (with discrimination > 2.0 considered substantial) and beta-thresholds that indicate the amount of visual disability a respondent would have to exhibit on the theta-ability continuum before answering one category higher on the Likert scale becomes more likely.^23^ Items showing poor discrimination or thresholds are removed from further consideration. GRM then uses the adjusted item discriminatory ability and difficulty calculations of retained items to impute new “visual disability” and cumulative factor scores, which were used for regression analyses to assess the association between various types of VI vs self-reported visual ability.

### Differential Item Functioning 

As part of the IRT analysis, a final check on psychometric purity was conducted by checking for item bias whereby item response is affected by incidental variables (e.g. demographics) other than the ability variable it is intended to measure exclusively.^25^ Uniform and nonuniform DIF analyses were used for this purpose (i.e. to ensure there were no remaining items that were biased by demographic covariates). We investigated DIF on six dichotomized demographic subgroups; age (+/− 75 years old), gender (female/male), education (any schooling/no schooling), type of home (fully paid/ partially or fully subsidized), depression (moderate-severe/mild), and diabetes (yes/no).

Uniform DIF assumes the item bias is in the same direction at all levels of the visual disability continuum, where one demographic subgroup might supposedly demonstrate a greater or lesser item score holding ability constant, compared to its demographic counterpart. Nonuniform DIF indicates whether there is significant dissimilarity in item score between the two subgroups, conditional on the disability level, reflected by demographic subgroup by ability interaction.^25^^,^^26^ Uniform DIF analyses were performed using linear models, where the individual item score represented the dependent variable, the demographic variable, the primary independent variable, and the estimated mean theta-ability score (calculated in IRT) represented the covariate. For nonuniform DIF, an additional interaction term for the cross-product of the theta score and the binarized demographic variable was added to the model to determine whether there was significant and substantial dissimilarity between demographic groups conditional on the level of disability. Any such significant interaction was explored post hoc within a range of one standard deviation from the mean theta disability level to determine the precise nature of the interaction (i.e. at what ability levels the groups differed on the item, and how).

### Statistical Analysis

Multivariable linear regression analyses of mean factor difficulty score were stratified by VI cause, and adjusted for age, gender, education, housing, diabetes, and depression. For all analyses, 95% confidence intervals for coefficients or other effect estimates are presented, and a *P* value of < 0.05 was considered statistically significant. Stata version 16 (StataCorp LP, College Station, TX) was used for all analyses.^27^

## Results

### Patient Characteristics

Of the 867 participants who completed the IND-VFQ-33 questionnaire, 683 had normal vision (78.7%; Table 1). Of those with VI (*n* = 184), 62 had an uncorrected refractive error (URE), 81 had cataract, and 41 had VI due to other causes. There was no substantial or significant difference in age or gender between different categories of VI, or between each cause of VI and no VI (*P* > 0.05 for all). All causes of VI were associated with significantly lower likelihood of education beyond high school versus no VI (*P* < 0.05 for all), and total VI was associated with a significantly lower likelihood of diabetes versus no VI (*P* < 0.05). There were no other significant differences in baseline demographic features between VI cause versus no VI (*P* > 0.05; Table 1).

**Table 1.** Baseline Demographic Features of Questionnaire Participants

	No Visual Impairment(*n* = 683)	Uncorrected Refractive Error (*n* = 62)	Cataract (*n* = 81)	Other Causes^a^ (*n* = 41)	Total Visual Impairment (*n* = 184)
**Age, mean (SD)**	74.0 (8.1)	73.8 (8.3)	75.0 (8.9)	77.1 (9.4)	75.0 (8.8)
**Female, n (%)**	424 (62.1)	39 (62.9)	49 (60.5)	25 (61.0)	113 (61.4)
**Education, n (%)**					
**- < High School**	70 (10.3)	14 (22.6)	20 (24.7)	12 (29.3)	46 (25)
**- High School**	448 (65.6)	40 (64.5)	46 (56.8)	25 (61.0)	111 (60.3)
**- > High School**	165 (24.2)	8 (12.9)	15 (18.5)	4 (9.8)	27 (14.7)
**Type of home, *n* (%)**					
**- Fully subsidized**	92 (13.5)	10 (16.1)	20 (24.7)	5 (12.2)	35 (19)
**- Partial subsidy**	303 (44.4)	30 (48.4)	34 (42.0)	20 (48.8)	84 (45.7)
**- Fully paid**	288 (42.2)	22 (35.5)	27 (33.3)	16 (39.0)	65 (35.3)
**- Diabetes, n (%)**	223 (32.7)	12 (19.4)	16 (19.8)	12 (29.3)	40 (21.7)
**Depression, n (%)**					
**- None-mild**	546 (79.9)	44 (71.0)	58 (71.6)	24 (58.5)	126 (68.5)
**- Moderate**	85 (12.5)	7 (11.3)	8 (9.9)	4 (9.8)	19 (10.3)
**- Severe**	52 (7.6)	11 (17.7)	15 (18.5)	13 (31.7)	39 (21.2)

a Other causes: including age-related macular degeneration, diabetic retinopathy, posterior capsule opacification.

Most of the 33 items in the questionnaire were comprehensively answered (< 5% missing data). Items with particularly high missing data were item 8 (difficulty seeing the step of a bus climbing in or out; 36.7% missing), item 4 (difficulty going to social functions like weddings; 32% missing), item 13 (difficulty doing work up to usual standard; 31.4% missing), item 1 (difficulty climbing stairs; 29.6% missing), and item 5 (difficulty finding way in new places; 23.4% missing), and were removed from further analyses.

### Psychometric Properties of the INDVFQ-33

#### Factor Analysis

Exploratory factor analysis and parallel analysis revealed two substantive factors that each demonstrated unidimensionality and had a unique constellation of loading items. The parallel analysis confirmed no further factors reliably assessed by the questionnaire. Fifteen items reliably loaded onto factor 1, associated clinically with difficulty in performing daily activities. Six items loaded reliably onto factor 2, associated clinically with visual symptoms (Table 2). Item 32, “does light seem like stars,” loaded particularly poorly onto either factor, as did items 9, 13, 25, 26, 27, and 33 (loading < 0.5); and were removed from subsequent analyses (Fig. 1). Items 1, 4, 5, 8, and 13 (above) all had particularly high missingness. To ensure consistency and comparability of the total information gained from items for the remainder of the analysis, these items (which provided substantially less total information) were removed from further analyses.

**Table 2.** Results of the Rotated (Promax Oblique) Factor Analysis, With Final Factor Loadings and IRT Discrimination and Difficulty Results

	Discrimination (CI)	B2 Threshold^a^	B3 Threshold	B4 Threshold	B5 Threshold	*P* Value
**Factor 1: Daily Activities** **^b^** ^,^ ^c^						
2. Making out bumps/holes in the road while walking?	3.13 (2.65–3.61)	0.55	1.40	1.70	2.64	< 0.001
3. Seeing if there are animals or vehicles when walking?	3.66 (3.02–4.29)	1.10	1.56	1.91	2.75	< 0.001
6. Going out at night?	2.43 (2.05–2.81)	0.79	1.51	1.77	2.19	< 0.001
7. Finding your way indoors?	4.44 (3.52–5.36)	1.34	1.94	2.28	3.28	< 0.001
10. Recognizing the face of a person standing near you?	3.54 (2.84–4.25)	1.43	2.05	2.39	2.82	< 0.001
11. Locking or unlocking the door?	3.69 (3.02–4.35)	1.19	1.80	2.19	2.53	< 0.001
12. Doing your usual work either in the house or outside?	3.81 (3.15–4.47)	1.08	1.64	2.00	2.81	< 0.001
14. Searching for things at home?	3.46 (2.90–4.02)	0.86	1.53	1.88	2.69	< 0.001
17. Seeing differences in colour?	2.64 (2.21–3.08)	1.02	1.82	2.07	2.67	< 0.001
18. Making out differences in coins or notes?	2.69 (2.28–3.11)	0.85	1.60	1.90	2.42	< 0.001
19. Going to the toilet?	3.44 (2.80–4.07)	1.26	1.86	2.17	3.46	< 0.001
20. Seeing objects that may have fallen in the food?	3.31 (2.77–3.86)	1.03	1.63	1.89	2.25	< 0.001
21. Seeing the level in the container when pouring?	3.20 (2.69–3.70)	0.96	1.63	1.92	2.46	< 0.001
23. Do you enjoy social functions less?	2.58 (2.11–3.05)	1.11	1.51	1.74	–	< 0.001
24. Are you ashamed that you can't see?	2.34 (1.90–2.78)	1.44	1.97	2.31	–	< 0.001
**Factor 2: Visual Symptoms**						
15. Seeing outside in bright sunlight?	2.26 (1.93–2.58)	0.30	1.36	1.73	2.81	< 0.001
16. Seeing when coming into the house after being in sunlight?	1.99 (1.71–2.27)	−0.52	1.32	1.83	3.00	< 0.001
28. Are you dazzled in bright light?	2.38 (2.03–2.74)	0.40	1.36	1.87	–	< 0.001
29. Is your vision blurred in sunlight?	3.48 (2.89–4.07)	0.18	1.26	1.67	–	< 0.001
30. Does bright light hurt your eyes?	2.81 92.37–3.26)	0.49	1.21	1.76	–	< 0.001
31. Do you close your eyes because of light from vehicles?	1.92 (1.64–2.20)	−0.43	0.91	1.30	–	< 0.001

Key:

a B value = theta value at which it becomes more likely for participant to choose one option higher (find task one increment harder) on the 4- or 5-point difficulty scale (where the B2 represents the theta ability level at which it becomes more likely for participant to select response 2 than response 1).

b Questions 2–21 begin with “Because of your vision how much problem do you have in….”

c Questions 22–31 begin with “Because of your eye problem….”

Cronbach alpha coefficient calculations concluded an alpha value of 0.95 for factor 1, and 0.88 for factor 2. Factors 1 and 2 were estimated to correlate at r = +0.79. Thus, although the analysis indicated the factors were statistically and conceptually internally cohesive and distinct from each other, they were still moderately positively correlated.

#### Item Response Theory

Table 2 presents the results of the IRT GRM analysis. All 15 remaining items loading onto factor 1 had a high discrimination (representative of good item ability to detect score differences by differences in level of visual ability) and all results reached statistical significance at the alpha = 0.05 level. All 6 remaining items loading onto factor 2 had a high discrimination of > 2.2, excluding items 16 and 31 (discrimination 1.99 and 1.92, respectively), which were retained. Similarly, all results reached statistical significance at the alpha = 0.05 level (Table 2).

The item difficulty parameters (beta thresholds, representative of the level on the theta ability continuum) reflect the range of underlying participant ability for each factor. The theta-ability level for factor 1 (daily activities) ranged from 0.55–3.46 across persons, and the theta-ability level for factor 2 (visual symptoms) ranged from −0.52 to 3.0. All questions within each factor showed good item-difficulty variability allowing good differentiation of participant ability for any given item. Beta-thresholds were used to scale item difficulty at different levels of ability.

### Differential Item Functioning

In total, 13 items demonstrated statistically significant uniform DIF. Of those items, no DIF was considered substantial enough between subgroups to consider item modification / removal (all < 0.29 difference in total item score, out of 5). Similarly, in nonuniform DIF analysis, there was no substantial dissimilarity in item score for all 16 items demonstrating significant nonuniform DIF (conditional on the level of disability) explored to within one standard deviation of the mean disability level. The “depression” demographic subgroup demonstrated the greatest heterogeneity, with predicted item score differential still within 0.5 out of a total of 5 points.

These results suggested that, when holding the level of disability constant, there was no significant and substantial difference in item score attributable to baseline characteristics (even when conditioning on the level of disability; i.e. no DIF item bias). Subsequently, no further item reduction was undertaken at this stage of the analysis.

### Regression Outcomes Using IRT-Adjusted Factor Scores

Participants with all forms of VI reported greater difficulty with factor 1 daily activities versus those with normal vision (Table 3). Participants with other causes of VI reported the highest level of difficulty discrepancy (9.4 points higher) and participants with URE the lowest level of difficulty discrepancy (2 points higher; *P* < 0.05 for all) versus those with normal vision. Higher education was independently associated with a lower degree of reported difficulty experienced for all causes of VI, and depression was independently associated with a higher degree of reported difficulty for all causes of VI (*P* < 0.05 for all; Table 3).

**Table 3.** Multivariable Linear Regression Demonstrating Difference in Expected Total Factor Score Compared to Those With Normal Vision, Stratified by VI Cause

	Uncorrected Refractive Error (*n* = 61)^a^	Cataract (*n* = 81)	Other Causes^b^ (*n* = 41)	Total Visual Impairment (*n* = 184)
**Factor 1: Daily Activities** ^c^				
	**Change in Score (CI)**	**Change in Score (CI)**	**Change in Score (CI)**	**Change in Score (CI)**
**Baseline (intercept)** ^d^	17.4 (13.4 to 21.5)	18.1 (13.6 to 22.7)	19.5 (14.8 to 24.1)	19.1 (14.4 to 23.8)
**Mean score change**	**+ 2.0 (0.5 to 3.5)**	**+ 7.6 (6.1 to 9.1)**	**+ 9.4 (7.4 to 11.5)**	**+ 6.0 (4.8 to 7.2)**
**Covariates**				
**- Age > 75 y**	0.0 (0.0 to 0.1)	0.0 (‒0.0 to 0.1)	0.0 (‒0.1 to 0.1)	0.0 (‒0.1 to 0.1)
**- Female**	0.1 (‒0.8 to 1.0)	0.1 (‒0.9 to 1.0)	‒0.4 (‒1.3 to 0.6)	‒0.6 (‒1.6 to 0.4)
**- Any education**	**‒2.1 (‒3.4 to ‒0.8)**	**‒2.9 (‒4.3 to ‒1.4)**	**‒2.4 (‒3.9 to ‒0.9)**	**‒3.2 (‒4.7 to ‒1.7)**
**- Fully paid**	**‒1.0 (‒1.8 to ‒0.1)**	‒1.0 (‒1.9 to 0.0)	‒0.8 (‒1.8 to 0.1)	**‒1.1 (‒2.1 to 0.0)**
**- Diabetes**	0.5 (‒0.4 to 1.4)	0.3 (‒0.7 to 1.3)	0.2 (‒0.8 to 1.2)	0.3 (‒0.8 to 1.4)
**- Depression**	**4.6 (3.6 to 5.6)**	**5.1 (4.0 to 6.2)**	**5.5 (4.3 to 6.7)**	**6.2 (5.0 to 7.3)**
**Factor 2: Visual Symptoms**				
**Baseline (intercept)**	10.3 (7.9 to 12.7)	10.6 (8.1 to 13.1)	11.5 (9.0 to 13.9)	11.0 (8.6 to 13.3)
**Mean score change**	**+ 1.1 (0.2 to 2.0)**	**+ 2.2 (1.4 to 3.0)**	**+ 1.9 (0.8 to 3.0)**	**+ 1.7 (1.1 to 2.3)**
**Covariates**				
**- Age > 75 y**	0.0 (0.0 to 0.0)	0.0 (0.0 to 0.0)	0.0 (0.0 to 0.0)	0.0 (0.0 to 0.0)
**- Female**	‒0.1 (‒0.7 to 0.4)	‒0.1 (‒0.7 to 0.4)	‒0.2 (‒0.7 to 0.3)	‒0.3 (‒0.8 to 0.2)
**- Any education**	**‒1.0 (‒1.8 to ‒0.2)**	**‒1.3 (‒2.1 to ‒0.5)**	**‒1.1 (‒1.9 to ‒0.3)**	**‒1.1 (‒1.9 to ‒0.4)**
**- Fully paid home**	**‒0.7 (‒1.3 to ‒0.2)**	**‒0.8 (‒1.3 to ‒0.2)**	**‒0.6 (‒1.1 to ‒0.1)**	**‒0.8 (‒1.3 to ‒0.3)**
**- Diabetes**	**0.6 (0.0 to 1.1)**	0.4 (‒0.1 to 1.0)	0.5 (‒0.1 to 1.0)	0.4 (‒0.1 to 0.9)
**- Depression**	**2.8 (2.2 to 3.4)**	**3.0 (2.4 to 3.7)**	**3.0 (2.4 to 3.7)**	**3.1 (2.5 to 3.7)**

a Those with severe VI 2’ URE (*n* = 1) were omitted from analysis. Subsequent URE *n* = 61 (rather than URE *n* = 62, as in Table 1).

b Other causes including age-related macular degeneration, diabetic retinopathy, and posterior capsule opacification.

c Factor 1 out of a possible 73 points; factor 2 out of a possible 26 points.

d Baseline (intercept): expected score for those without any visual impairment.

All results held constant for: age (reference < 75), female (reference male), any education (reference none), independently paid housing (referenced subsidized), depression (reference none), and diabetes (reference none).

Significant covariates displayed in bold.

Unstandardized partial regression coefficients shown.

Participants with all types of VI also reported greater difficulty with factor 2 visual symptoms versus those with normal vision (Table 3). Participants with cataract reported the highest level of difficulty discrepancy (2.2 points higher) and participants with URE the lowest level of difficulty discrepancy (1.1 points higher; *P* < 0.05 for all) versus those with normal vision. Higher education and paid housing (a measure of greater wealth) were independently associated with a lower degree of reported difficulty experienced for all causes of VI, and (similarly to daily activities) depression was independently associated with a higher degree of reported difficulty for visual symptoms, for all causes of VI (*P* < 0.05 for all; Table 3).

**Figure 2.**
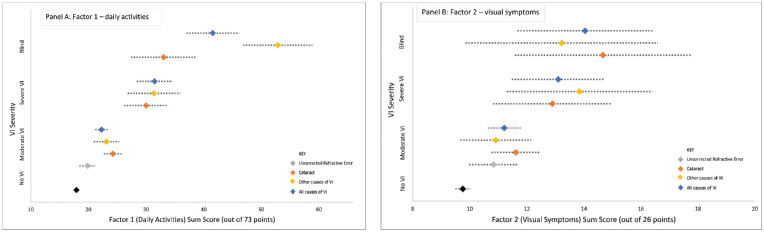
Multivariate IRT-adjusted factor 1 (daily activities) and factor 2 (visual symptoms) sum score with 95% confidence intervals, by VI cause and severity.

Cause-specific analysis for factor 1 daily activities demonstrated greater self-reported difficulty associated with higher levels of VI for all forms of vision loss (Table 4, Fig. 2). Compared to those with normal vision, individuals with severe VI or blindness secondary to any cause demonstrated greater difficulty than moderate VI secondary to any cause (+ 13.4 and + 23.5 points, respectively, versus + 4.2 points, *P* < 0.001 for all). Compared to subjects with normal vision, individuals with severe VI or blindness secondary to cataract both demonstrated greater difficulty than moderate VI secondary to cataract (+ 12.1 points and + 15.1 points, respectively, versus + 6.3 points, *P* < 0.001 for all). In addition, compared to those with normal vision, individuals with severe VI or blindness secondary to other causes both demonstrated greater difficulty than moderate VI secondary to non-cataract causes (+ 13.5 points and + 34.9 points, respectively, versus + 5.2 points, *P* < 0.001 for all).

**Table 4.** Multivariable Linear Regression Demonstrating Difference in Expected Total Factor Score Compared to Those With Normal Vision, Stratified by VI Cause and Severity

	Uncorrected Refractive Error (*n* = 61)^a^		Cataract (*n* = 81)		Other Causes (*n* = 41)^b^		Total Visual Impairment (*n* = 184)	
**Factor 1: Daily Activities** **^c^**								
	**Change in Score (95% CI)**	***P* Value**	**Change in score (95% CI)**	***P* Value**	**Change in score (95% CI)**	***P* Value**	**Change in score (95% CI)**	***P* Value**
**VI severity**								
**Normal**	Reference	–	Reference	–	Reference	–	Reference	–
**Moderate**	+ 2.0 (0.5–3.5)	0.01	+ 6.3 (4.7–8.0)	< 0.001	+ 5.2 (2.9–7.5)	< 0.001	+ 4.2 (3.0–5.4)	< 0.001
**Severe**	*None* ^d^	–	+ 12.1 (8.2–15.9)	< 0.001	+ 13.5 (8.9–18.0)	< 0.001	+ 13.4 (10.3–16.6)	< 0.001
**Blind**	*None*	–	+ 15.1 (9.5–20.8)	< 0.001	+ 34.9 (28.9–41.0)	< 0.001	+ 23.5 (18.9–28.1)	< 0.001
**Factor 2: Visual Symptoms**								
**VI severity**								
**Normal**	Reference	–	Reference	–	Reference	–	Reference	–
**Moderate**	+ 1.1 (0.2–2.0)	0.02	+ 1.9 (1.0–2.8)	< 0.001	+ 1.2 (-0.1 to 2.4)	0.07	+ 1.4 (0.8–2.0)	< 0.001
**Severe**	*None^a^*	–	+ 3.1 (1.0–5.3)	0.003	+ 4.1 (1.5–6.7)	0.002	+ 3.3 (1.6–4.9)	< 0.001
**Blind**	*None*	–	+ 4.9 (1.8–8.0)	0.002	+ 3.5 (0.1–6.9)	0.04	+ 4.2 (1.8–6.6)	0.001

a Those with severe VI 2’ URE (*n* = 1) were omitted from analysis. Subsequent URE *n* = 61 (rather than URE *n* = 62, as in Table 1).

b Other causes including age-related macular degeneration, diabetic retinopathy, and posterior capsule opacification.

c Factor 1 out of a possible 73 points; factor 2 out of a possible 26 points.

d Those with severe VI 2’ URE (*n* = 1) omitted from analysis. Subsequent URE *n* = 61 (rather than URE *n* = 62, as in Table 1).

Similarly, cause-specific analysis for factor 2 visual symptoms also demonstrated greater self-reported difficulty associated with higher levels of VI, except for the category “other causes” (Table 4, Fig. 2). Compared to those with normal vision, individuals with severe VI or blindness secondary to any cause demonstrated greater difficulty than moderate VI secondary to any cause (+ 3.3 points and + 4.2 points, respectively, versus + 1.4 points, *P* < 0.001 for all). Compared to subjects with normal vision, individuals with severe VI or blindness secondary to cataract both demonstrated greater difficulty than moderate VI secondary to cataract (+ 3.1 points and + 4.9 points, respectively, versus + 1.9 points, *P* < 0.003 for all). In addition, compared to those with normal vision, individuals with severe VI or blindness secondary to other causes both demonstrated greater difficulty than moderate VI secondary to non-cataract causes (+ 4.1 points and + 3.5 points, respectively, versus + 1.1 points, *P* < 0.05 for all; Fig. 2).

## Discussion

VI was associated with substantially higher self-reported functional difficulty in both daily activities and visual symptoms in an elderly institutionalized Indian population. Although all causes of VI result in functional difficulty, VI secondary to cataract and other ocular disease conferred greater level of difficulty compared to URE. Additionally, more severe cataract and ocular disease was associated with greater vision-related functional disability.

The association between VI and difficulty performing visual and physical tasks in an elderly, residential care population in India has not been previously described. General community populations in India have shown poorer self-reported function performing visual-related tasks, and a lower quality of life, with VI due to any cause, particularly for those with age-related cataract and glaucoma; findings which have notably persisted even when adjusting for presenting visual acuity.^28^^,^^29^ This is congruent with the findings of the present study. Previous studies have also demonstrated that as visual acuity severity increases, so too does the level of self-reported functional difficulty, congruent with present findings.^28^^,^^29^

Importantly, our study describes that those with cataract-related VI reported the highest level of difficulty in both factors (daily activities and visual symptoms) whereas those with uncorrected refractive error reported substantially lower levels of difficulty above baseline (albeit still more difficulty than those without VI). This is consistent with similar studies of similar populations, which have described cataract as independently associated with poor visual function holding presenting visual acuity constant,^28^^,^^29^ and more debilitating than other causes of VI; emphasizing potential functional benefits of cataract surgery in such populations.^30^^,^^31^ Notably, URE has been previously described as poorly related to self-reported visual function and quality of life in similar populations.^29^^,^^32^ This is partially congruent with current findings of only a marginal increase in reported functional difficulty for patients with URE compared to those with no VI. This may be in part due to the better presenting visual acuity in patients with URE versus cataract, as reported elsewhere^29^; although even when stratifying by presenting VA severity, patients with URE still reported lower difficulty from baseline versus patients with cataract. Another possible explanation is that because URE may be more long-standing than cataract, patients have learned to functionally compensate and do not report as much subjective functional restriction. However, length of diagnosis data was not available for the present study. A third possible explanation is that URE may result in worse quality of distance vision when compared to that of cataract. In any case, our findings point to the important benefit of cataract extraction in improving subjective visual function and likely quality of life, which have been described elsewhere in detail.^33^^–^^35^

Additionally, we found that depression was independently associated with self-reported visual difficulty, irrespective of the cause of VI. The relationship between depression and VI is complex, and likely bidirectional.^36^ Although it could be inferred that impaired visual acuity and subsequent visual difficulty leads to depression; it could equally be inferred that depression itself worsens any existing difficulty performing vision-dependent tasks.^36^^–^^39^ This finding points to an important relationship among VI, visual function, and depression that has been previously well described.^40^^–^^43^ Given that depression alone is a known independent cause of functional disability, independent of VI,^44^^,^^45^ our findings corroborate previous findings associating depression with VI. Findings further suggest that eye care providers and primary health care providers should be particularly aware of the association between depression and vision loss (especially among the elderly) and offer appropriate eye care services and/or referral to patients exhibiting depressive symptoms, earlier in management. For example, tools like the PHQ-9 depression screening questionnaire might be used to screen patients with VI for depression and refer if necessary.

Education and residence in “paid/private” homes, which were found to be “protective” against self-reported visual difficulty, may represent a surrogate measurement of economic status, and resource availability. It is possible that as well as being able to afford residential care independently, these residents may also have better access to eye care and subsequently experience a lower degree of visual difficulty. We found a lower prevalence of VI and self-reported visual difficulty in these groups compared to those living in “free” homes.^2^

A unique element of the present study was the use of factor analysis, IRT, and DIF to psychometrically validate the IND-VFQ-33 questionnaire, which distinguishes the present study from others comparing the association of VI and functional difficulty. The GRM chosen for the current study is particularly well-suited for psychometric validation of ordinal-scaled Likert-type questionnaires like the IND-VFQ-33 for a number of reasons.^46^^,^^47^ The GRM is less constrained than other psychometric models (i.e. Rasch or Partial Credit models), imposes fewer assumptions (i.e. equal discrimination) and fewer restrictions on the data,^48^ and consequently allows for a more realistic reflection of which latent traits are being measured, and how well each question contributes toward that measurement,^49^ formulating rescaled patient difficulty scores for accurate between-group comparison particularly when using larger datasets as in the present study. In the present study, two clinically distinct latent traits were identified with collections of questions that corresponded strongly and uniquely to each trait. Further, DIF analysis allowed us to conclude that visual impairment was the primary factor driving the magnitude of self-reported difficulty, and that there were no other baseline demographic variables that substantially influenced this.

The present study is strengthened by its large sample size with a relatively good response rate for most questionnaire items. The clinical assessments and interviews were done in residential homes to ensure comfort and convenience for elderly participants, and psychometric validation techniques undertaken allowed accurate calculation of modified visual difficulty scores and better insight into the degree of a subjective functional burden for elderly patients. Limitations to the study include its generalizability, given the population studied was exclusively residential-care based. This may in part explain why five of the original questions had such low response rates, given they pertained to activities rarely done by someone living in residential aged care, representing potential information bias. Indeed, the IND-VFQ-33 had been previously validated in a general Indian population living of all ages in the community, so the modified version demonstrated here may be particularly suited to residential aged-care populations only. Finally, as with all data of a self-reported nature, there is a potential for reporter bias.

Cataract and uncorrected refractive error were the major cause of VI in the present study, both of which can be addressed with relatively cost-effective interventions. The current research highlights the major functional impact of VI on this population lending further support to establishing a more systematic approach to identifying VI and addressing it in the elderly in residential care in India.
